# Pseudotyping the adenovirus serotype 5 capsid with both the fibre and penton of serotype 35 enhances vascular smooth muscle cell transduction

**DOI:** 10.1038/gt.2013.44

**Published:** 2013-09-05

**Authors:** A L Parker, K M White, C A Lavery, J Custers, S N Waddington, A H Baker

**Affiliations:** 1 British Heart Foundation Glasgow Cardiovascular Research Centre, College of Medical, Veterinary and Life Sciences, University of Glasgow, Glasgow, UK; 2 Crucell BV, Archimedesweg,Leiden, The Netherlands; 3 Gene Transfer Technology Group, Institute for Women's Health, University College London, London, UK

**Keywords:** vascular smooth muscle cell, adenovirus, CD46 receptor, coxsackie and adenovirus receptor, neointima, coronary artery bypass grafting

## Abstract

*Ex vivo* gene therapy during coronary artery bypass grafting (CABG) holds great potential to prevent excessive smooth muscle cell (SMC) proliferation, neointima formation and graft failure. The most successful preclinical strategies to date have utilised vectors based on the species C adenovirus, Ad5, which engages the Coxsackie and Adenovirus receptor (CAR) as its primary attachment receptor. Profiling receptors on human SMCs demonstrated the absence of CAR but substantial expression of the species B receptor CD46. We performed transduction experiments using Ad5 and the CD46-utilising adenovirus Ad35, and found Ad35 significantly more efficient at transducing SMCs. To evaluate whether transduction could be further augmented, we evaluated chimeric CD46-utilising Ad5/Ad35 vectors comprising the Ad5 capsid pseudotyped with the Ad35 fibre alone (Ad5/F35) or in combination with the Ad35 penton (Ad5/F35/P35). In human smooth muscle cells (hSMCs), Ad5/F35/P35 mediated significantly higher levels of transduction than either parental vector or Ad5/F35. *Ex vivo* transduction experiments using mouse aortas from CD46 transgenics demonstrated that Ad5/F35/P35 was significantly more efficient at transducing SMCs than the other vectors tested. Finally, *ex vivo* transduction and immunofluorescent colocalisation experiments using human tissue from CABG procedures confirmed the preclinical potential of Ad5/F35/P35 as an efficient vector for vascular transduction during CABG.

## Introduction

Cardiovascular disease remains the leading cause of mortality worldwide, accounting for approximately one-third of global deaths annually.^1^ A leading cause of cardiovascular disease is the development of atherosclerotic plaques within the coronary arteries. Where the plaque is stable, the condition may remain asymptomatic for some time until the resulting vessel occlusion becomes significant enough to restrict blood flow, depleting oxygen and nutrient deliveries and resulting in angina pectoris. When unstable, these plaques may rupture, resulting in thrombosis and myocardial infarction. Coronary artery bypass grafting (CABG) has been the mainstay clinical procedure to alleviate the symptoms of acute coronary symptoms, whereby a peripheral section of vasculature is utilised as a conduit to bypass the occluded vessel. Although arterial conduits can be used for this procedure (most commonly the left and the right internal mammary arteries), normally autologous sections of saphenous vein are utilised.

Following CABG, the grafted vessel undergoes a process of remodelling its phenotype to adapt to the increased pressure and shear rates of the arterial circulation. To achieve this, the surrounding smooth muscle cell (SMC) layer proliferates and expands to increase vessel plasticity. However, excessive migration and proliferation of the SMC layer can result in vessel reocclusion (restenosis) and ultimately graft failure. Around 25% of all grafts fail within the first year because of thrombotic occlusion (∼10%) or intimal thickening (∼15%), with a further 25% failing within 10 years because of accelerated atherosclerosis.^2^ Consequently, genetic modulation of the vasculature to overexpress antiproliferative genes may have therapeutic benefit in preventing vein graft failure in patients undergoing CABG. During the bypass procedure, the grafted vessel is available outside the body for ∼30 min prior to grafting, thus providing an opportunity for *ex vivo* gene therapy. A number of potentially therapeutic antiproliferative genes have undergone preclinical evaluation and shown promise for their capacity to limit SMC proliferation and neointima formation, including p53,^3^ NOGO-B^4^ and TIMP-3.^5, 6, 7^ To date, studies have focussed primarily on the use of serotype 5 adenovirus (Ad5), a species C adenovirus, to achieve overexpression of therapeutic transgene within the vasculature;^4, 6, 7^ however, uptake of Ad5 across the vessel wall and the resulting level of gene transfer mediated through Ad5 is relatively poor and necessitates very high input titres (typically >10^10^ pfu per graft). Additionally, a significant proportion of patients present pre-existing neutralising antibodies against Ad5.^8, 9, 10^ Collectively, these suboptimal characteristics of Ad5 could limit the progression and interpretation of vascular gene therapy in the clinic. Efficacy could be improved through identification and development of more efficient adenovirus-based vectors that efficiently transduce the vasculature at lower, less toxic input doses. We have therefore evaluated the expression of known primary adenoviral receptors on cultures of human smooth muscle cells (hSMCs) in order to rationally develop more efficacious vectors for vascular gene-transfer applications. On the basis of our findings that Coxsackie and Adenovirus receptor (CAR), the Ad5 receptor,^11^ is not expressed on hSMCs, we have focussed our attention on Ad35-based vectors, as CD46, the species BI Ad35 receptor,^12^ is expressed at high levels on hSMCs. We therefore evaluated a panel of CD46-interacting Ad5/Ad35 chimeric vectors for their potential for vascular gene-therapy applications using cells in culture and *ex vivo* gene transfer to the vasculature. Our findings uncover a potentially important and previously undocumented role for the Ad35 penton in enhancing transduction of the vasculature, which may have important translational applications for CABG.

## Results and Discussion

**Figure 1:**
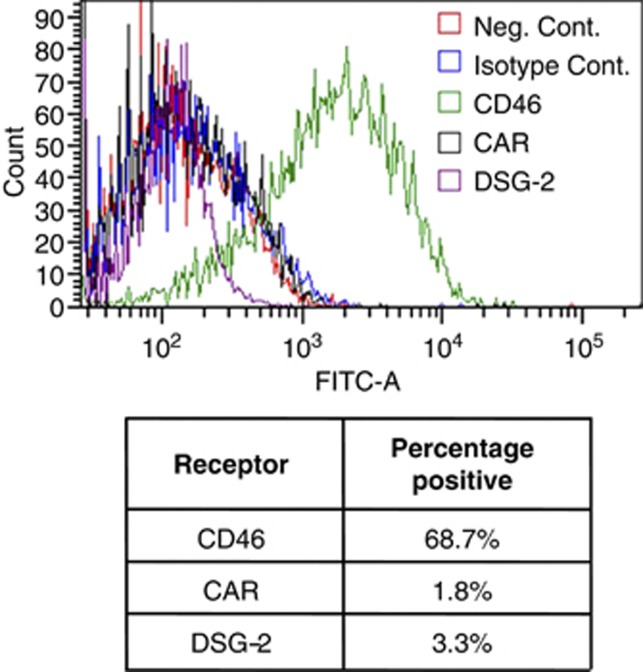
FACS analysis to profile expression of adenoviral receptors on cultures of hSMCs. Cultures of hSMCs were stained for the expression of CAR (black), CD46 (green) and desmoglein-2 (purple) using the antibodies RmcB, MEM-258 and 6D8, respectively. IgG and unstained controls are also shown (blue and red, respectively). Receptor expression was averaged across three samples and quantified.

A number of previous publications have demonstrated that the CAR-utilising species C adenovirus, serotype 5, can be efficient at transducing vascular SMCs when deployed at very high titres (typically >1 × 10^10^ pfu per graft).^13^ At lower doses, the cells are relatively refractory to Ad5 infection. We therefore sought to evaluate whether this could be improved upon utilising alternative species of adenovirus that utilise alternative receptors. In order to rationally develop adenoviral vectors with improved vascular transduction capabilities, we first screened cultures of hSMC to quantify the expression levels of known adenoviral receptors on the cell surface by fluorescence-activated cell sorting (FACS). Surprisingly, we found very low levels of CAR expression on all cultures of hSMC cells tested, with <2% of cells staining positively for CAR expression (Figure 1). In contrast, we found substantial expression of the species B receptor, CD46, in cultures of hSMC cells tested, varying from 60 to 100% of cells staining positively for expression. We also evaluated levels of expression of the recently identified species BII receptor, Desmoglein-2, and found that, like CAR it was not expressed on hSMC.

**Figure 2:**
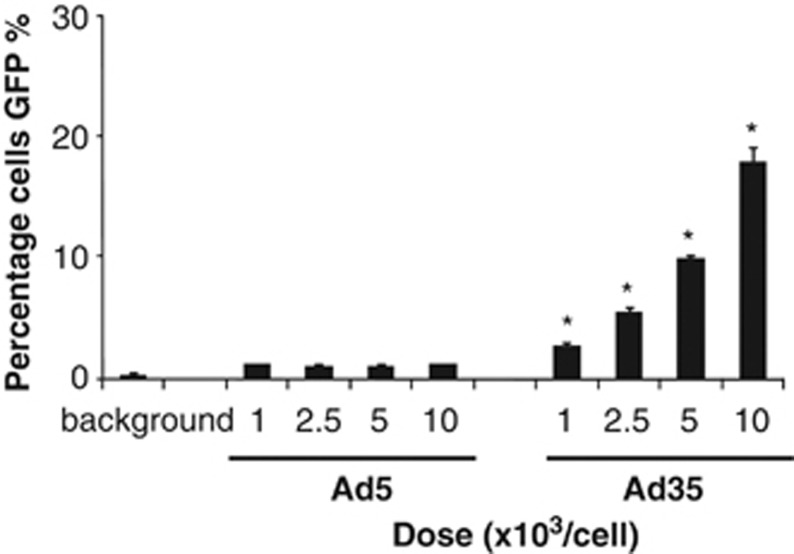
Transduction of hSMCs with Ad5 and Ad35 vectors. Cultures of hSMCs were plated to a density of 20 000 cells per well in 96-well plates and infected with 1000–10000 vp of Ad5 or Ad35 expressing eGFP. Forty-eight hours post infection, eGFP expression was quantified using FACS. Error bars represent s.d. of three individual samples, *P<0.01 versus Ad5.

Given the differential expression levels of adenoviral receptors on primary cultures of hSMC, we rationalised that vectors based on species B, CD46-interacting adenoviruses might be more efficacious for vascular gene-transfer applications than CAR-utilising vectors based on Ad5. We first performed transduction experiments in cultures of hSMC using enhanced green-fluorescent protein (eGFP) expressing Ad5 and Ad35 at a range of doses from 1000 to 10 000 vp per cell. FACS analysis of transduced cells 48 h post infection demonstrated that at all doses tested, very few hSMC cells were positive for eGFP expression when Ad5 was used (Figure 2). However, when Ad35-eGFP was utilised, we observed a dose-dependent increase in eGFP expression, up to ∼20% of cells were positive at the highest dose tested.

**Figure 3:**
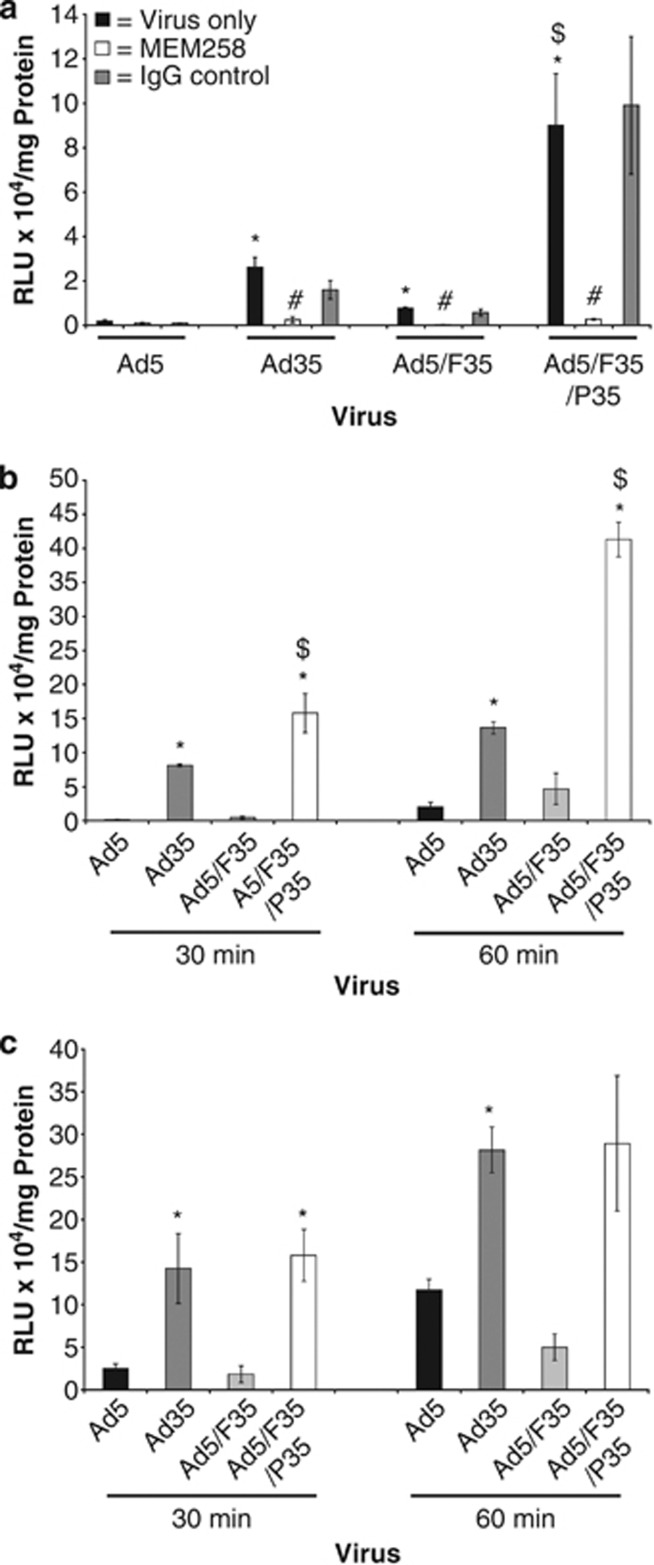
Transduction of hSMCs and human endothelial cells (hECs) using Ad5, Ad35 and chimeric Ad5/Ad35 vectors. Cultures of hSMC were plated at a density of 20 000 cells per well in 96-well plates. Cells were then incubated with CD46-blocking antibody MEM-258 or IgG control for 1 h on ice, before being infected with 1000 vp per cell of Ad5, Ad35, Ad5/F35 or Ad5/F35/P35 expressing luciferase for 1 h. After 1 h, media was removed; the cells were cultured for a further 48 h in complete media before the analysis of luciferase transgene expression was performed. *P<0.01 versus Ad5, ^$^*P<*0.01 versus Ad35, ^#^*P<*0.01 versus IgG-blocking control (**a**). Cultures of hSMCs (**b**) or hECs (**c**) were transduced with 1000 vp per cell of Ad5, Ad35, Ad5/F35 or Ad5/F35/P35 for 30 min or 1 h, before media was removed; the cells were cultured for a further 48 h in complete media before the analysis of luciferase transgene expression was performed. *P<0.05 increased versus Ad5, ^$^*P<*0.05 increased versus Ad35. Error bars represent s.d. of three individual samples.

Given the increased efficiency of vectors based on CD46-interacting Ad35 in transducing hSMC, we next evaluated whether chimeric Ad5/Ad35 vectors could enhance vascular transduction further. We have previously described a panel of luciferase-expressing adenoviral vectors comprising the parental vectors Ad5 or Ad35, or the Ad5 capsid pseudotyped with either the Ad35 fibre alone (Ad5/F35) or in combination with the penton of Ad35 (Ad5/F35/P35), their interactions with blood-clotting factor X, and their cell tropism and biodistribution in CD46-transgenic mice.^14^ In order to assess for any additive effects of the chimeric vectors over the parental forms in transducing hSMCs, we utilised the same panel of vectors. To our surprise, we found that Ad5/F35/P35 reproducibly mediated higher levels of transduction in hSMC vectors compared with either of the parental vectors or Ad5/F35 (Figure 3a). We confirmed that transduction of Ad35, Ad5/F35 and Ad5/F35/P35 was dependent on CD46 by preincubating cells with the CD46-blocking antibody (MEM-258). The increased transduction we observed using Ad5/F35/P35 appeared to be a selective phenomenon for hSMCs, as the same enhancements were not observed in cultures of human endothelial cells (Figure 3c), which demonstrated similar levels of transduction as Ad35, similarly to several CD46-positive cancer cell lines evaluated (data not shown), in agreement with our previous observations.^14^ Furthermore, we demonstrate that these increased levels of transduction occur within a clinically relevant time frame of 30 or 60 min (Figure 3b (hSMCs) and 3c (human endothelial cells)) and that, as expected, levels of transduction increased with increasing exposure periods.

**Figure 4:**
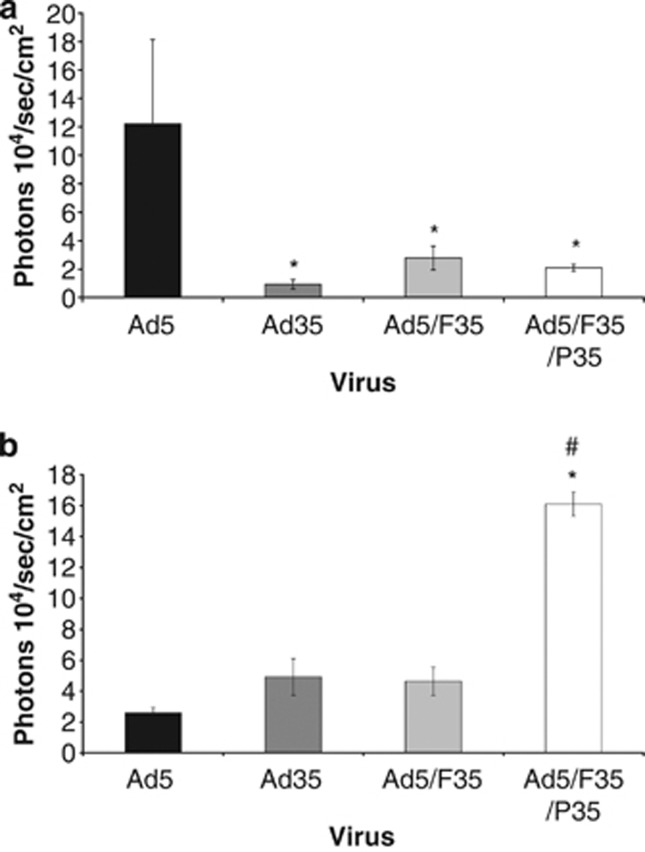
*Ex vivo* transduction in intact mouse vasculature. Aortas were isolated from MF1 mice (**a**) or CD46-transgenic mice (**b**) and divided into three equally sized pieces. The tissue was then incubated in 500 μl of serum-free media containing 1 × 10^9^ vp of Ad vector. After 1 h of incubation, tissue was cultured for a further 48 h. The vessels were then subject to bioluminescence quantification imaging, with transduction expressed as average radiance (p s^−1^ cm^−2^ sr^−1^). *P<0.05 versus Ad5, ^#^*P<*0.05 versus Ad35.

To investigate whether the increased transduction efficiency we observed in cultures of hSMCs could be recapitulated in intact vessels with polarised cells, we performed *ex vivo* transduction experiments using aortas isolated from wild-type (MF1) or CD46-transgenic mice. Aortas were isolated and transduced using 1 × 10^9^ vp of luciferase-expressing Ad5, Ad35, Ad5/F35 or Ad5/F35/P35. In wild-type, MF1 mice, which do not express CD46, except in the gonads,^15^ Ad5 mediated the highest levels of transduction, significantly higher than Ad35 or either chimeric vector (Figure 4a). However, in CD46-transgenic mice on an MF1 background,^16^ Ad5/F35/P35 mediated approximately four times higher levels of transduction than Ad35 or Ad5/F35, and approximately eight-fold higher transduction than Ad5, confirming the importance of the presence of CD46 for the uptake and transduction of Ad35-based vectors (Figure 4b).

**Figure 5:**
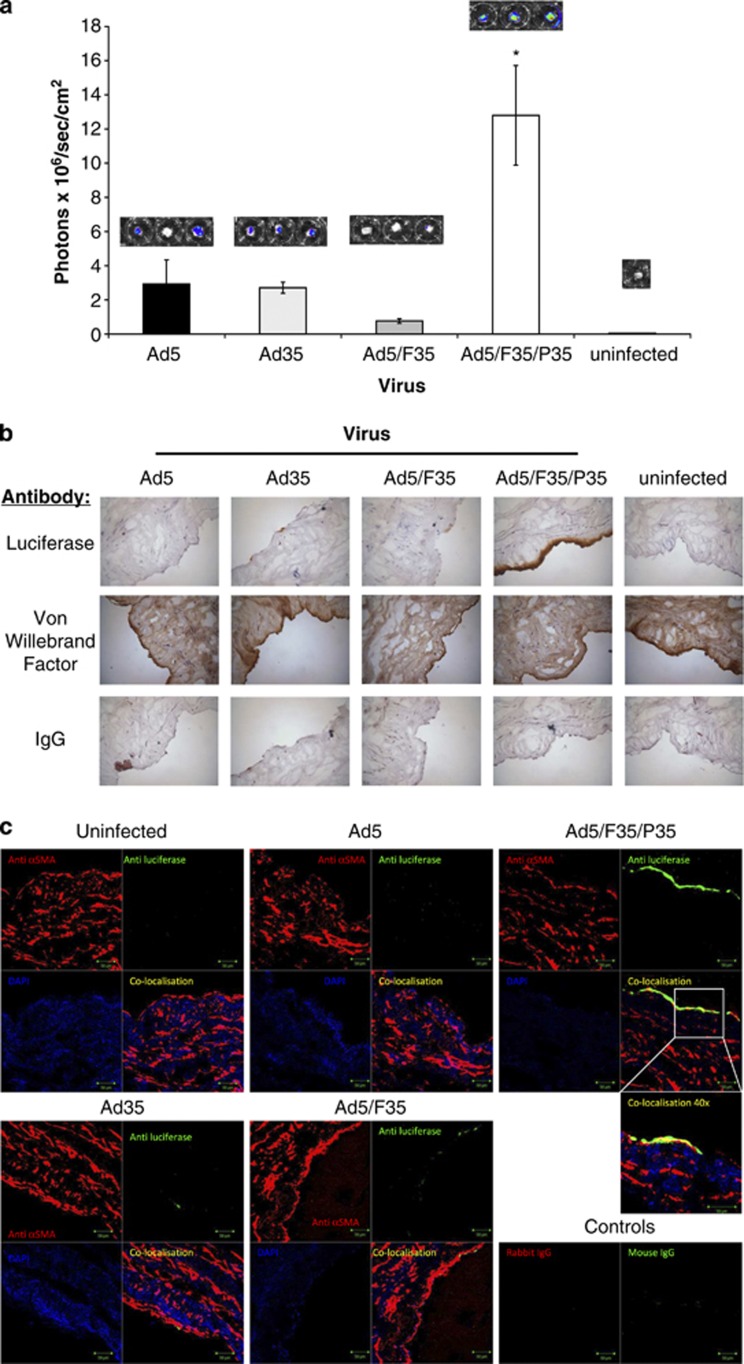
*Ex vivo* transduction in the intact human saphenous vein. Saphenous vein from bypass grafting procedures were divided into sections of 5–6 mm in length and incubated in 500 μl of serum-free media containing 1 × 10^10^ vp of Ad vector. After 1 h of incubation, tissue was cultured for a further 48 h in complete media. The vessels were then subjected to bioluminescence quantification imaging, with transduction expressed as average radiance (p s^−1^ cm^−2^ sr^−1^) (**a**). *P<0.01 versus Ad5. Luciferase expression was detected histologically in frozen sections using a luciferase primary antibody (or IgG control) and a HRP-conjugated secondary antibody, and staining compared with von Willebrand factor (to detect endothelial cells). Representative images are shown, magnification × 40. (**b**) Luciferase expression (green) was detected histologically in frozen sections using a luciferase primary antibody (or IgG control), and colocalistion (yellow) was determined with the smooth muscle cell marker, α-smooth muscle actin (red). Representative images are shown, magnification × 25 (except zoomed image, × 40) (**c**).

As a preclinical model for therapeutic gene delivery to the vasculature, we obtained excess human saphenous vein tissue from CABG procedures. Samples were transduced in triplicate for 1 h at 37 °C with luciferase-expressing Ad5, Ad35, Ad5/F35 or Ad5/F35/P35. After 48 h, luciferase expression within the vessel was quantified. Consistent with the data obtained in cultured hSMCs and in CD46-transgenic mouse vasculature, we observed significantly higher levels of transduction with Ad5/F35/P35 compared to the other vectors (Figure 5a). Coincidentally in this model, Ad5 mediated broadly similar levels of transduction to Ad35 (approximately four-fold lower than Ad5/F35/P35), despite their differing receptor usage and expression levels of CAR and CD46. To assess transgene localisation, we performed immunohistochemical staining on the vessels. Immunohistological analysis of the transduced vessels (Figure 5b) clearly demonstrated the superiority of Ad5/F35/P35 in transducing the vessel *per se*. To confirm expression in SMCs, an important parameter for smooth muscle-targeted therapies, we performed immunofluorescent colocalisation experiments for luciferase and α smooth muscle actin (Figure 5c). We observed colocalised expression in the SMC layer in vessels transduced with Ad5/F35/P35 but not in control vessels or those transduced with Ad5, Ad35 or Ad5/F35 (Figure 5c).

Following CABG, the grafted vessel (normally derived from the saphenous vein) remodels in order to cope with the elevated pressure and shear rate of the arterial circulation compared with the lower pressure of the venous circulation from where the vessel was derived (reviewed in McDonald *et al.*^17^) The remodelling process necessitates the migration and proliferation of vascular smooth muscle cells to increase vessel plasticity; however, this may result in neointima formation and subsequent vessel reocclusion. Vessel reocclusion following engraftment represents a major limitation of bypass graft surgery, contributing significantly to a cumulative graft failure rate of ∼50% within 10 years post surgery.^2^ A potential means to limit proliferation and thus prevent graft failure is by performing *ex vivo* gene therapy to deliver antiproliferative genes to the conduit during the grafting procedure (overviewed in Robertson *et al.*^18^). To this end, we have demonstrated potential for a number of therapeutic antiproliferative genes including NOGO-B,^4^ p53^3, 19^ and TIMP-3^6, 7^ in preventing vessel reocclusion using preclinical models in mice and pigs. To achieve therapeutic levels of transgene expression, we have utilised high-concentration adenoviruses based on serotype 5. *In vitro*, and for local gene-therapy applications *in vivo*, Ad5 binds to cells predominantly through the primary interaction with the CAR^11, 20^ and internalisation through integrin engagement.^21^ We have found that efficient transduction of the vasculature requires very high input of Ad5, typically >10^10^ pfu per graft. Given the potential for dose-limiting toxicities at such high doses and the very high levels of pre-existing immunity against Ad5 in the general population, we reasoned that the development of more efficacious vectors that transduce the vasculature efficiently at lower doses during the clinical window of opportunity for *ex vivo* gene therapy during bypass grafting may be advantageous in the clinical setting.

Our observation that cultures of hSMC express vanishingly low levels of the Ad5 receptor CAR may explain the requirement for extremely high doses of Ad5 to efficiently transduce hSMCs. It is possible that at such high titres, engagement of secondary receptors, namely the integrin αvβ3 and αvβ5, which are expressed at high levels on hSMCs,^2^ may mediate the uptake of Ad5. We observed similarly low levels of the recently identified species BII receptor desmoglein-2, the cellular receptor for Ad3, 7, 11 and 14.^3^ Given the high levels of expression of the species B receptor CD46 observed in all cultures tested, we rationalised that adenoviral vectors based on CD46-interacting species of adenovirus might be better suited to translational CABG gene-transfer applications. We therefore focussed our study on the potential of Ad35, a serotype well known to utilise CD46 as its primary cellular receptor,^12, 24, 25^ to deliver reporter genes to cultures of hSMCs *in vitro*. At all doses tested, we found that Ad35 was more efficient at transducing hSMCs than Ad5. Evaluating the effects of capsid pseudotyping on transduction efficiency of cultured hSMCs demonstrated that all viruses pseudotyped with the Ad35 fibre transduced cells in a CD46-dependent manner. Unexpectedly, we noted that the additional pseudotyping of the Ad35 penton into the Ad5 capsid had a beneficial effect on transduction, enhancing transduction of hSMCs by greater than three-fold and approximately ten-fold relative to Ad5/F35. Interestingly, this enhancing effect on transduction appeared selective for SMCs, as transduction experiments in cancer cell lines showed no additional augmentation of transduction (data not shown).

Given that CD46 is expressed at high levels on cultures of hSMCs (and that CAR is not), and the additional beneficial effects on transduction of pseudotyping the Ad5 capsid with both the fibre and penton from Ad35, we performed preclinical studies using intact vessels. This is potentially important because de-differentiation of cells *in vitro* is a well-characterised phenomenon,^27, 28^ and *in vitro* culture systems are often unable to reproduce the polarised phenotype of cells *in vivo*. This may result in potentially promising *in vitro* results failing to reproduce *in vivo,* as the receptors are not accessible from the appropriate compartment (that is, the luminal surface) in intact vessels. Systems that work well *in vitro* but fail through polarisation and inaccessibility of receptors *in vivo* are well known in gene therapy.^29^ We therefore performed studies in intact mouse vessels from wild-type (MF1) and CD46-transgenic mice, as murine expression of CD46 is limited to the testes,^15^ whereas CD46 is expressed ubiquitously in the human. In aortas derived from MF1 mice, Ad5 mediated significantly higher levels of transduction than any of the CD46-utilising Ads. However, in the CD46-transgenic mouse, we observed the highest levels of transduction using Ad5/F35/P35, consistent with our *in vitro* observations, whereas both Ad35 and Ad5/F35 mediated significantly higher levels of transduction than Ad5.

Furthermore, in *ex vivo* transduction experiments using excess residual clinical material from coronary artery bypass procedures, we again noted that Ad5/F35/P35 mediated higher levels of transduction than any other virus tested, mediating approximately four-fold higher transduction than either parental vector Ad5 or Ad35, gauged by IVIS imaging. Importantly, as the vessel comprises multiple cell types, we confirmed the presence of transgene expression in SMCs using immunofluorescence and demonstrated that Ad5/F35/P35 was the only vector that efficiently transduced this cell type at the doses tested.

Taken together, our data suggest that vectors based on species B, CD46-interacting adenoviruses likely hold greater potential for translational CABG gene-therapy applications than species C, CAR-interacting adenoviruses. We also identify an additional role for the Ad35 penton in enhancing transduction of Ad5/F35-pseudotyped vectors in hSMCs; however, the precise role of the penton in this context remains unclear but likely relates to improved uptake (compared with Ad5) and intracellular trafficking (compared with Ad35) in hSMCs.

## Materials and methods

### Cell culture

Human SMCs and ECs were prepared using residual tissue from CABG procedures as described previously.^30, 31^ Informed consent and ethics approval were obtained prior to this procedure. SMCs were maintained in SMC growth media 2 (Promocell, Heidleberg, Germany) containing 15% fetal calf serum (FCS) and passaged for a maximum of six times. ECs were maintained in Large Vessel Endothelial Cell Basal Medium (TCS CellWorks, Buckingham, UK) containing 20% FCS. Other cell lines utilised were cultured in RPMI media containing 10% FCS and maintained at 37 °C and 5% CO_2_.

### FACS analysis of adenoviral receptors expressed on hSMCs

Cells were resuspended to a density of 2 × 10^5^ cells per tube in 50 μl of cold serum-free media and incubated with 50 μl SF media containing 1/250 v/v (final concentration thus 1/500) of mouse antihuman monoclonal antibody against CAR (RmcB, Millipore, Watford, UK), CD46 (MEM-258, Abcam, Cambridge, UK) Desmoglein-2 (6D8, Hycult biotech, Uden, The Netherlands) or mouse IgG control antibody (Invitrogen, Paisley, UK) for 30 min on ice. Cells were washed in 4 ml PBS and centrifuged (1000 r.p.m., 5 min, 4 °C). PBS was discarded, and cells were incubated with 1:250 dilution of goat anti-mouse Alexa Fluor 488 (Invitrogen) secondary antibody in serum-free media for 30 min on ice. Alexa Fluor-488 fluorescently labelled cells were detected using a FACS Canto II flow cytometer (Beckton Dickinson, Oxford, UK) and FACS DIVA software. Viable cells were gated on the basis of forward and side scatter profiles, with a minimum of 10 000 gated events analysed per sample. Results are expressed as percentage of positive cells per condition. For each condition, a minimum of three separately treated samples were analysed and an average was taken.

### Adenovirus production, purification and quality control

All viruses described in this manuscript are first-generation, replication-incompetent E1-deleted vectors, propagated in the E1-complementing cell line 293 cells. Virus was purified using standard CsCl gradients and kept for long-term storage in 10% Glycerol, and 90% TE buffer. Viral recovery was quantified by microBCA assay using the following equation: 1 μg protein=4 × 10^9^ viral particles (vp). Infectious units (pfu) were quantified by end point dilution plaque assay on 293 permissive cells using the standard protocols. Physical particle titres for Ad5, Ad35, Ad5/F35 and Ad5/F35/P35 were 2.70 × 10^12^, 8.00 × 10^12^, 5.07 × 10^12^ and 2.70 × 10^12^, respectively, whereas infectious particle titres were 2.60 × 10^10^, 4.77 × 10^10^, 1.50 × 10^10^ and 2.95 × 10^9^, respectively, resulting in vp:pfu ratios of 104, 168, 338 and 915, respectively. As toxicity *in vivo* is determined by the physical particle load rather than infectious units, we standardise all our experiments to vp.

### *In vitro* transduction

A total of 2 × 10^4^ cells per well were washed with PBS before the addition of 1000–10000 vp per cell Ad-expressing luciferase in 50 μl of serum-free media for 3 h at 37 °C. Media were discarded and replaced with complete media and incubated at 37 °C for a further 45 h. Cell lysate was generated by the addition of 100 μl 1 × reporter lysis buffer (RLB, Promega, Warrington, UK) per well, followed by freeze/thawing prior to the analysis of reporter gene activity. Luciferase activity in the resulting lysate was quantified by the addition of 100 μl luciferase assay substrate (Promega) to 10 μl of cell lysate diluted with 90 μl RLB. Luciferase activity was quantified using a Wallac VICTOR2 plate reader (PerkinElmer Life and Analytical Sciences, Boston MA, USA) and normalised to total protein content of the samples, measured by bicinchoninic acid assay as per the manufacturer's instructions, producing relative light units per milligram protein (RLU mg^−1^ protein).

### *Ex vivo* transduction of vessels

Mouse aorta were isolated from either MF1 wild-type mice or CD46-transgenic on an MF1 background and divided into three equally sized pieces. The tissue was washed in PBS and incubated in 500 μl of serum-free media containing 1 × 10^9^ vp of vector. After 1 h of incubation, media was discarded and cultured in EMEM containing 20% FCS for a further 2 days. For studies involving intact human vessel, excess material from CABG procedures was trimmed to remove excess periadventitial fat and was divided into equally sized sections of ∼5 mm width and placed into a 24-well plate. The vessel was incubated with 500 μl of serum-free media containing 1 × 10^10^ vp of vector. After 1 h of incubation, the media containing virus was discarded, then tissue was washed with PBS and cultured in SMC growth media containing 15% FCS for a further 2 days at 37 °C and 5% CO_2_. Following 48 h in culture, the transduced vessels were subject to bioluminescence quantification imaging (IVIS Spectrum, Caliper Life Sciences, Hopkinton, MA, USA). Luciferin activity was measured using the IVIS Imaging System (IVIS Spectrum, Caliper), and transduction is expressed as average radiance (photons per second per centimetre squared per steradian (p s^−1^ cm^−2^ sr^−1^)).

### Immunohistological/immunofluorescent detection of luciferase expression in human vessels

Immunohistochemistry was performed on 6 μm frozen sections with mouse anti-luciferase antibody (Luci17; Santa Cruz Biotechnology, Heidelberg, Germany), rabbit anti-von Willebrand Factor (A0082; Dako, Glostrup, Denmark) or the appropriate IgG control (Dako). Sections were fixed with ice-cold acetone and then treated with 3% hydrogen peroxide to inhibit endogenous peroxidase activity. Sections were blocked in 10% serum and then treated with avidin/biotin kit (Vector Laboratories, Peterborough, UK). For detection, the appropriate biotinylated secondary antibody (Dako) and ExtrAvidin-Peroxidase (SigmaAldrich, Suffolk, UK) were used. Staining was visualised using 3',3'-diaminobenzidine tetrahydrochlorate dihydrate and hydrogen peroxide. Sections were counterstained with haematoxylin.

Immunofluorescence was performed on 6 μm frozen sections with mouse anti-luciferase antibody, rabbit anti-α smooth muscle actin (ab5694; Abcam) or the appropriate IgG control (Dako). Sections were fixed with ice-cold acetone, and then antigen retrieval was performed by heating in 10 mM sodium citrate buffer (pH 6). Sections were blocked in 10% goat serum. For detection, goat anti-mouse alexa546 and goat anti-rabbit alexa647 (Invitrogen) were used. Slides were mounted in ProLong Gold Antifade Reagent with DAPI (Invitrogen). Images were acquired using a Zeiss LSM510 confocal imaging system using LSM image acquisition software (Carl Zeiss, Welwyn Garden City, UK).

### Statistics

Results presented are representative data from a minimum of three separate experiments with at least three experimental replicates per group, unless otherwise stated. Statistical significance was calculated using two-sample, two-tailed Student's *t-*tests. *P<*0.05 was considered statistically significant.
